# Analysis of DNA Methylation and Hydroxymethylation in the Genome of Crustacean *Daphnia pulex*

**DOI:** 10.3390/genes7010001

**Published:** 2015-12-31

**Authors:** Dovilė Strepetkaitė, Gediminas Alzbutas, Eimantas Astromskas, Arūnas Lagunavičius, Rasa Sabaliauskaitė, Kęstutis Arbačiauskas, Juozas Lazutka

**Affiliations:** 1 Department of Botany and Genetics, Vilnius University, 21 Čiurlionis Str., LT-03101 Vilnius, Lithuania; dovile.strepetkaite@thermofisher.com; 2 Thermo Fisher Scientific Baltics, Graičiūno g. 8, LT-02241 Vilnius, Lithuania; gediminas.alzbutas@thermofisher.com (G.A.); eimantas.astromskas@thermofisher.com (E.A.); arunas.lagunavicius@thermofisher.com (A.L.); rasa.sabaliauskaite@thermofisher.com (R.S.); 3 Nature Research Centre, Akademijos str. 2, LT-08412 Vilnius, Lithuania; arbas@ekoi.lt

**Keywords:** *Daphnia pulex*, 5-methyl-cytosine, 5-hydroxymethyl-cytosine, whole genome sequencing, epigenetic modifications, *Cox4*, *Cand2*, *Ephx1*

## Abstract

The aim of our study was to analyze the presence of 5-methyl-cytosine (5-mC) and 5-hydroxymethyl-cytosine (5-hmC) in the genome of crustacean *Daphnia pulex*. First, the presence of 5-mC and 5-hmC in genomic DNA was demonstrated by using antibodies specific to either 5-mC or 5-hmC. Then, analysis of 5-mC and 5-hmC using pairs of restriction enzymes with different sensitivity to methylation and hydroxymethylation confirmed the presence of both modifications in selected regions of three genes (*Cox4*, *Cand2* and *Ephx1*). To get a detailed picture of 5-hmC distribution over the *D. pulex* genome, we performed 5-hmC enrichment and sequenced the enriched fraction using next generation sequencing and non-enriched library (input) as a control. Comparison of input and enriched libraries showed that 5-hmC in exons is twice as frequent as in introns. Functional analysis indicated that 5-hmC abundance is associated with genes that are involved in the adenylate cyclase-activating G-protein-coupled receptor signaling pathway, molting cycles, morphogenesis and cell fate determination. Genes that lack 5-hmC tend to be involved in the regulation of the transforming growth factor beta receptor signaling pathway and in many mRNA-related processes. Our results suggest that epigenetic modifications are present in the genome of *D. pulex* and most likely are involved in the regulation of gene expression of this crustacean.

## 1. Introduction

Daphnids are freshwater crustaceans that, dependent on environmental conditions, can reproduce either sexually or parthenogenetically. Water flea *Daphnia pulex* propagates by cyclical parthenogenesis producing subitaneous eggs. With deterioration of environmental quality, usually at the end of the growing season, these animals initiate sexual reproduction which results in two diapausing eggs encased in ephippium, a protective structure modified from carapace [1,2]. Emergence from diapausing eggs in daphnids takes place in the early season during a relatively short period [3,4,5], although these eggs, being resistant to external factors, can remain viable for extended time periods [6]. In addition, *D. pulex* exhibit different polyphenisms [7,8]. Since clonal lines are genetically identical but consist of phenotypically divergent individuals, this phenomenon could be attributed to the epigenetic changes [9,10].

Due to the relatively recently published genome sequence of *D. pulex* [11], the genome of this organism now is one of the most intensively studied among aquatic invertebrates. However, despite much encouragement [9,10], investigations of the epigenome of *Daphnia* are scarce. The presence of 5-methylcytosine was shown in the genome of *D. magna* [12,13]. Bioinformatic analysis indicated that *D. pulex* do have DNA methyltransferases as well [14], and potentially can methylate their genome; however, direct evidence is still lacking.

Until a few years back only one epigenetic DNA modification was well known—5-methylcytosine (5-mC). This modification has been extensively studied and a number of important epigenetic functions (e.g., gene regulation, X chromosome imprinting) are known. In 2009, 5-hydroxymethylcytosine (5-hmC) was rediscovered, resulting in a new age of epigenetics [15]. The modification of 5-hmC immediately became intensively studied and subsequent studies revealed the mechanism of producing this base *in vivo* via TET1-mediated oxidation [16]. While there are no TET oxygenases described in *D. pulex*, a recently published bioinformatic search suggested some candidates [17].

The aim of our study was to analyze the presence of 5-mC and 5-hmC in the genome of *D. pulex* using different methods, such as immuno-dot blot analysis, next generation sequencing (NGS) and digestion of genomic DNA with several pairs of restriction enzymes with different sensitivity to methylation and hydroxymethylation.

## 2. Experimental Section

### 2.1. Preparation of Biological Samples

A cyclically parthenogenetic population of *D. pulex* from a permanent pond in Vilnius was the source of animals for investigation [18]. The species was identified by analyzing both females and males. Clones of *D. pulex* were established from ephippia (winter eggs) that were collected in the pond after the ice cover had melted. In our laboratory, they were placed into trays filled with 0.45 μm membrane-filtered pond water and kept at 16 °C under permanent illumination. Exephippial hatchlings born during the two days were utilized for investigation and initiation of parthenogentic generation.

Exephippial hatchlings for the first three days of their life were raised in 200 mL volume vessels with ~40 individuals per vessel. Since the third day of their life, density of experimental animals was reduced to 20 individuals per vessel, and since maturation, the density was reduced to 10 specimens per vessel.

Parthenogenetic hatchlings were initiated from the second clutch of exephippial mothers. Before the clutch release, females were individually transferred into separate vessels. Each group of offspring of 40 specimens per vessel was composed by taking one hatchling from each 40 random clutches, thus these groups were mixtures of unique genotypes. Further, parthenogenetic offspring were raised in the same way as exephippial hatchlings.

Both morphs (exephippial and parthenogenetic) of *D. pulex* were raised at 20 °C under permanent illumination in membrane-filtered pond water under high food conditions, *i.e.*, daily provision of *Scenedesmus quadricauda* ~2.0 mg/L. Before cropping, animals were kept in filtered water overnight to get their gut clear. Further, daphnids were transferred to distilled water and counted, then were filtered on 0.5 mm mesh size net, rinsed with distilled water, inspected under microscope, transferred into microcentrifuge tubes and immediately frozen in liquid nitrogen. Samples were preserved at −70 °C until analysis.

For the analysis, animals of both morphs were cropped at the four ontogenetic stages: three-day-old juvenile stage (juveniles, 40 specimens per sample), five-day-old preadult animal stage (preadults, 20 specimens per sample) which, in this species, corresponds to instar 4 [19], about 12-day-old female carrying the second clutch stage (adults I, 10 specimens per sample), and over 15-day-old female carrying the third-fourth clutch stage (adults II, 10 specimens per sample). All these samples for both exephippial and parthenogenetic *D. pulex* morphs were composed of multiple clones. For next generation sequencing, samples consisting of single clone parthenogentic females, mostly adults, were used.

### 2.2. Preparation of Genomic DNA

Genomic DNA was purified using GeneJET^TM^ Plant Genomic DNA Purification Mini Kit (Thermo Scientific^TM^, Vilnius, Lithuania, protocol A) following manufacturer’s instructions. Concentration of purified DNA was measured using Nano Drop 2000 (Thermo Scientific) following manufacturer’s instructions.

DNA integrity was assessed in 1% agarose gel (TopVision™ agarose (Thermo Scientific), 50× TAE buffer (Thermo Scientific), 0.05 mg/mL ethidium bromide (Thermo Scientific). DNA samples were mixed with 6× DNA Loading Dye (Thermo Scientific).

### 2.3. Detection of Global Genome Methylation and Hydroxymethylation Using Immuno-Dot Blot Analysis

Immuno-dot blot was conducted using Biotin Chromogenic Detection Kit (Thermo Scientific) following manufacturer’s instructions. Primary 5-mC antibody (Active Motif, cat. no. 61255) was diluted 1000 times and primary 5-hmC antibody (Active Motif, cat. No. 39770) was diluted 10,000 times. Secondary rabbit antibody (Active Motif) in 5-hmC sample was diluted 2000 times and secondary mouse antibody (Active Motif) in 5-mC sample was diluted 5000 times. Total genomic methylation and hydroxymethylation levels were assesed by using TotalLab software, signal intensity was normalized according to amount of DNA used (genomic DNA of *D. Pulex*—200 ng, methylated and unmethylated DNA of the plasmid pUC—100 ng, human genomic DNA—625 ng, 5-hydroxymethylated DNA of *Staphylococcus aureus*—250 ng).

### 2.4. Local Methylation and Hydroxymethylation Analysis in Selected Genes of D. pulex

Local DNA modifications were analyzed in genomic regions located in three genes (Table 1): cullin-associated NEDD8-dissociated protein 2 (*Cand2*), cytochrome c oxidase, subunit IV (*Cox4*), and juvenile hormone epoxide hydrolase 1 (*Ephx1*). Primers for qPCR analysis (Table 2) were designed using NCBI PRIMER-BLAST [20] in such way that amplicons are covering both 5'-CCGG-3' and 5'-TCGA-3' sites in exonic regions. Primers with ΔG bigger than −2 kcal/mol were chosen

**Table 1:** Target genes and their parameters.

Gene	Gene Symbol	Genomic Location	Length, bp	Number of Exons	Length of Exons, bp
Juvenile hormone epoxide hydrolase 1	*Ephx1*	scaffold_121: 159299–164657	5358	11	1598
Cullin-associated Nedd8-dissociated 2	*Cand2*	scaffold_4446: 1087–2177	1090	4	882
Cytochrome c oxidase, subunit IV	*Cox4*	scaffold_23: 1334549–1335729	1180	4	780

**Table 2:** qPCR primer sequences for *D. pulex* genes of interest.

Gene	Primer	Primer Sequence
*Cox4*	Forward	AGTTGGAGACCCAGTTAAAGC
	Reverse	AGGTTTGGCAGAAAGATGCTC
*Cand2*	Forward	GAAATACTTGCACCGCCAGAG
	Reverse	TACTCCTGCAGCATTTCCGTG
*Ephx1*	Forward	CTCAAAACCCAGTGGGGAGG
	Reverse	TTGTCGGATTCTTGAGTCAGC

In order to evaluate the methylation level of target genes, 100 ng purified genomic DNA was treated by EpiJET^TM^ DNA Methylation Analysis Kit (MspI/HpaII) and (TaqI/HpyF30I) (Thermo Scientific) following manufacturer’s instructions. In order to assess target-gene hydroxymethylation level 100 ng purified genomic DNA was treated by EpiJET 5-hmC Analysis Kit (Thermo Scientific) following manufacturer’s instructions. qPCR was performed using 2× Maxima™ SYBR Green qPCR Master Mix (Thermo Scientific) following manufacturer’s instructions. Percent methylation/hydroxymethylation level at the target-gene site was evaluated according to formula: 100/(1 + E%)^(CT average of restriction endonuclease treated DNA-CT average of non-treated DNA), where E% is qPCR efficiency, calculated by StepOne Plus™ (Applied Biosystems, Carlsbad, CA, USA) from a standard curve, consisting of three DNA dilutions. All results were obtained from at least four replicates.

### 2.5. Next Generation Sequencing

For next generation sequencing, DNA from parthenogenetic *D. pulex* adults was used. Prior to DNA extraction, individuals from single clone were treated with Sephadex beads to clean the gut and 500 mg/l of tetracycline to reduce bacterial contamination, exactly as described by Colbourne *et al.* [11]. Then 100 ng of extracted DNA was fragmented using MuSeek^TM^ Library Preparation Kit, Illumina compatible (Thermo Scientific) and purified using GeneJET NGS Cleanup Kit (Thermo Scientific). Libraries for 5-hmC enrichment analysis were end-repaired, purified and 5-hmC was enriched using EpiJET 5-hmC Enrichment Kit (Thermo Scientific) according to manufacturer’s protocol. The enriched and non-enriched control (input) were PCR-amplified for 14 cycles and gel size selected for next generation sequencing. The libraries were analyzed using Agilent Bioanalyzer (Agilent) and KAPA library quantification kit (KAPA biosystems). The next generation sequencing for *de novo* assembly was performed using Illumina MiSeq platform with MiSeq reagent kit v3 600-cycle (Illumina). Libraries for 5-hmC analysis were sequenced using v3 150-cycle kit. The sequencing data is deposited in NCBI’s Sequence Read Archive. Reads for the reference genome assembly are deposited as SRR2968969 entry, reads of the two runs of the 5-hmC enriched library are deposited as SRR2970595 and SRR2970600.

### 2.6. NGS Data Analysis

#### 2.6.1. Initial Processing of Reads

Initially, the reads were processed using Cutadapt 1.8.1 [21]. Shorter-than-70-bp reads were discarded and the 3ʹ end of reads were trimmed to have quality value higher or equal to 20.

#### 2.6.2. Assembly of Genome

Genome assembly was done in two steps:

1. Reads that matched to draft *D. pulex* genome [11] were collected using Kraken 0.10.5 [22]. The overlapping 3' ends of reads were merged using FLASH v1.2.11 [23]. The assembly was performed using Spades 3.5.0 [24]. Bacterial contigs were filtered out using Kraken 0.10.5 and the remaining contigs were used for the subsequent step.
2. The overlapping 3ʹ ends of reads were merged using FLASH v1.2.11. Then they were assembled using Spades 3.5.0 together with the contigs from the first step. Contigs that matched to draft *D. pulex* genome [11] were collected using Kraken 0.10.5. Out of these contigs the bacterial ones were discarded using second run of filtering with Kraken 0.10.5.

In this way, prepared assembly was used to evaluate sequencing of some control libraries after 5-hmC enrichment. However, the distribution of 5-hmC across gene body and its association with potential gene function were analyzed after discarding reads with exceptionally high coverage (higher than 50× coverage). The statistics about assemblies were collected using QUAST 3.1 [25].

#### 2.6.3. Detection of Genes and Their Functional Annotation

Genes were detected using GlimmerHMM-3.0.4 [26], which was trained on already published *D. pulex* genome [11]. Functions of the detected genes were inferred (GO terms assigned) using PANNZER (Protein ANNotation with Z-scoRE) and its database (v.1.0) [27].

#### 2.6.4. The 5-hmC Peak Calling and Analysis

Before alignment reads were processed using Cutadapt 1.8.1. Shorter-than-35-bp reads were discarded and the 3ʹ end of reads were trimmed to have quality value higher or equal to 20. Reads were aligned to contigs using Bowtie2 v2.1.0 [28]. Peaks were detected using MACS2 v2.0.10 [29] allowing at most three duplicate reads. The distribution of peaks across the genome was analyzed using RSeQC v2.5 [30], BEDTools [31] and custom Python scripts. For functional analysis we selected two groups of genes: (1) genes having exons completely void of peaks; (2) genes having a density of peaks across gene body equal or higher than 10 PPKM (peaks per kilobase of transcript per million of peaks). Coordinates of protein coding exons for each gene were predicted with GlimmerHMM-3.0.4 [26]. PPKM is analogous to RPKM (reads per kilobase per million) measure that is used in RNA sequencing. PPKM measure normalizes counts of peaks to the length of protein-coding DNA and allows us to compare exons’ hydroxymethylation in genes of different lengths. The PPKM cut off was arbitrarily adjusted to have approximately same number of genes in both groups. The enrichment of all GO terms of these genes was evaluated between these two groups. Only those GO terms were considered for analysis which were detected in both groups. For further analysis we selected only the GO terms of which frequency was significantly different. The *p*-value 0.01 was used in cases of genes that had 5-hmC and for genes that lacked the peaks the *p*-value cut-off was 0.05. The selected GO terms were analyzed and visualized using REViGO [32].

## 3. Results and Discussion

**Figure 1:**
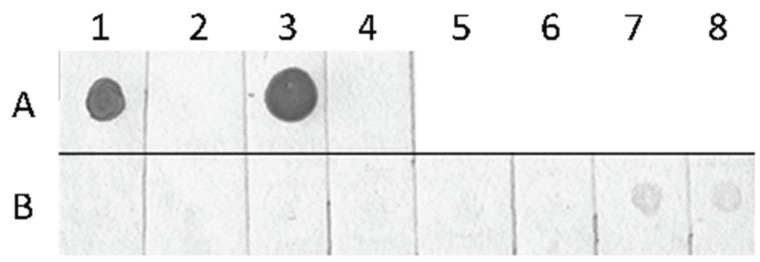
Immuno-dot blot using antibody specific for 5-methylcytosine. Lane A represents 100 ng of methylated DNA from the plasmid pUC (A1), 100 ng of unmethylated DNA from pUC (A2), 625 ng of human genomic DNA (A3), 250 ng of 5-hydroxymethylated DNA from *Staphylococcus aureus* (A4). Lane B—200 ng of genomic DNAs from *D. pulex* juveniles (B1, B2), preadults (B3, B4), adults I (B5, B6), and adults II (B7, B8); even numbers—individuals hatched form parthenogenetic eggs; odd numbers—individuals hatched from ephippial eggs.

The presence of cytosine methylation and hydroxymethylation in *D. pulex* genomic DNA was first assessed by immunological reaction, using antibodies specific for either 5-mC or 5-hmC. Immuno-dot blot using antibody specific for 5-mC showed a very weak signal only at a dot corresponding to the DNA of the oldest individuals (Figure 1). In younger individuals no signal was detectable, which could be due to a very low amount of 5-mC that is below the sensitivity of the immuno-dot blot assay.

**Figure 2:**
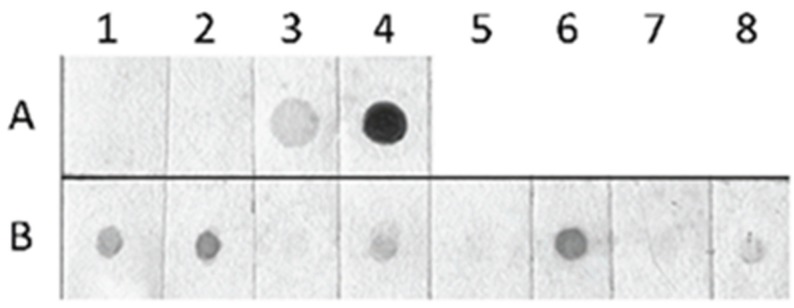
Immuno-dot blot using antibody specific for 5-hydroxymethylcytosine. Lane A represents 100 ng of methylated DNA from the plasmid pUC (A1), 100 ng of unmethylated DNA from pUC (A2), 625 ng of human genomic DNA (A3), 250 ng of 5-hydroxymethylated DNA from *Staphylococcus aureus* (A4). Lane B—200 ng of genomic DNAs from *D. pulex* juveniles (B1, B2), preadults (B3, B4), adults I (B5, B6), and adults II (B7, B8); even numbers—individuals hatched form parthenogenetic eggs; odd numbers—individuals hatched from ephippial eggs.

A different picture was seen in the case of immuno-dot blot using antibody specific for 5-hmC (Figure 2). A relatively strong signal was seen in dots with DNA from juveniles, preadults and adults I, and a only weak signal in dots with DNA from adults II. In all cases, variation between two morphs (hatched from winter and parthenogenetic eggs) of each ontogenetic stage could be noticed (in dots B3, B5 and B7 no signal was detectable).

As it was mentioned above, no direct measurements of methylation and hydroxymethylation levels in *D. pulex* have been published until now. However, DNA methylation was assessed in close species—*D. magna*, where by analyzing two genomic fragments the authors [12] determined relatively low methylation level—from 0.22% to 0.44% methylated CpG sites. Our data demonstrate, for the first time, the presence of both 5-mC and 5-hmC in the genome of *D. pulex*. A weak signal of 5-mC is in a good agreement with bioinformatics findings of other authors [14], where the profile of evolutionary CpG depletion in protein-coding genes of *D. pulex* suggests existence of DNA methylation in this crustacean. However, we feel that the immune-dot blot assay is not sensitive enough for such kinds of studies. It also does not allow any reliable quantitative analysis, since in invertebrates DNA modifications could occur in non-CpG context [33] and crude DNA preparations may contain significant amounts of DNA from other species, including various endosymbionts [34]. Unfortunately, more precise methods such as HPLC or MS analysis of total DNA do not discriminate the origins of DNA modifications, making the results of such analysis unreliable as well, and while the bisulphite sequencing will overcome the contaminating DNA issue, it will not discriminate between 5-mC and 5-hmC, thus providing no additional information about the distribution of 5-hmC over the genome of *D. pulex*. Keeping these reasons in mind, we decided to perform additional analysis, including digestion of genomic DNA with several pairs of restriction enzymes with different sensitivity to methylation and hydroxymethylation followed by locus-specific qPCR and total 5-hmC enrichment/NGS analysis.

**Figure 3:**
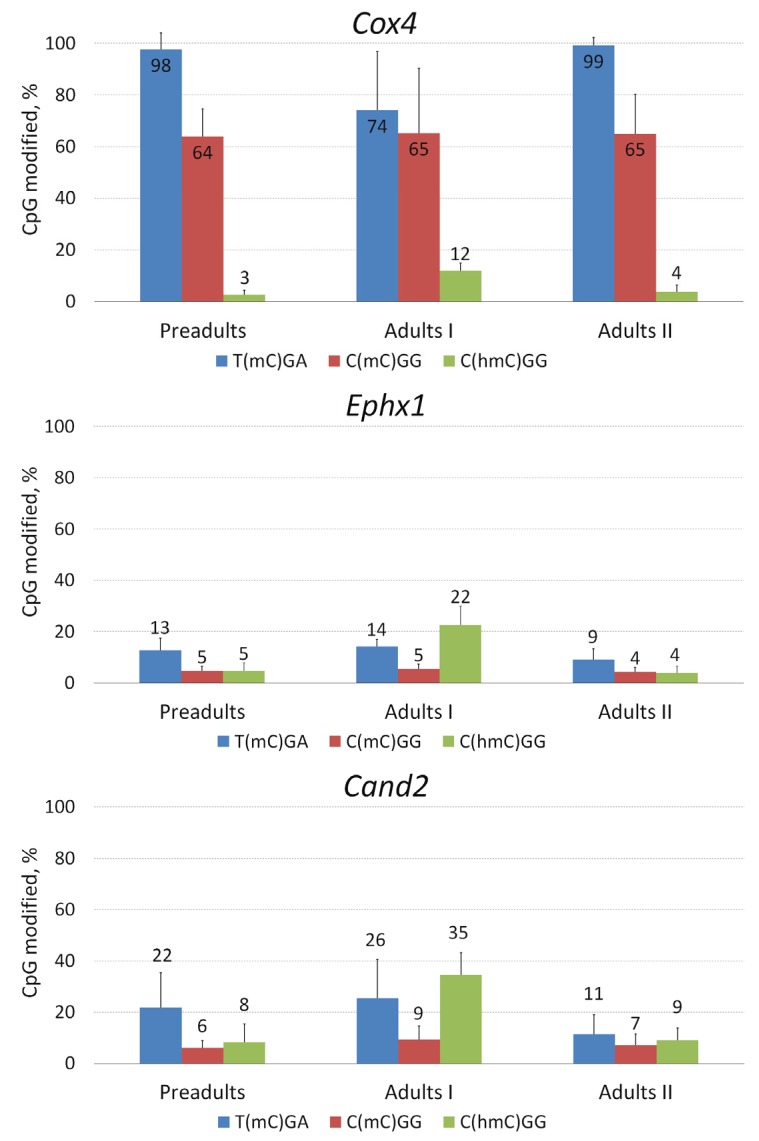
Percentage of CpG-modified sites in selected regions of three *D. pulex* genes—*Cox4*, *Ephx1* and *Cand2*. Blue columns represent percentage of methylated CpG in TCGA sequences, red columns—percentage of methylated CpG in CCGG sequences, green columns—percentage of hydroxymethyated CpG in CCGG sequences. Error bars represent standard deviation.

Thus, in our next set of experiments, we analyzed methylation and hydroxymethylation in regions of selected genes of *D. pulex* (Figure 3). For this analysis we used pairs of restriction endonucleases MspI/HpaII, which could detect methylation of the internal CpG in the CCGG tetranucleotide, and HpyF30I/TaqI, which could detect methylated cytosine in the TCGA tetranucleotide. First, 5-hmC was assessed in CCGG sites using T4 phage β-glucosyltransferase, which is capable of specifically modifying 5-hmC residues by adding a glucose moiety to 5-hmC, and restriction enzyme Epi MspI, the activity of which is completely blocked when 5-hmC is glucosylated.

Three genes that could be associated with the development of different morphs in *D. pulex* were chosen for the analysis. These were the cullin-associated NEDD8-dissociated 2 (*Cand2*) gene, cytochrome C oxidase subunit IV (*Cox4*) gene and juvenile hormone epoxide hydrolase 1 (*Ephx1*) gene. The *Cand2* gene has not been annotated in the genome of *D. pulex*; however, a few orthologs are known in other species, *Drosophila melanogaster* in particular. It has been demonstrated that the product of this gene interacts with trancription factor TBP and promotes myogenesis [35]. *Cox4* is a cytochrome c oxidase subunit responsible for the assembly of the whole cytochrome c oxydase [36]. It has been previously shown that *Cox4* is differently expressed in post-diapause individuals of *Artemia franciscana* [37] and in different morphs of *Acyrthosiphon pisum* [38]. Juvenile hormone epoxide hydrolase 1 (*Ephx1*) participates in the catabolism of the juvenile hormone and seems to be a key enzyme in juvenile hormone degradation [39].

In selected regions of the *Cox4* gene, high levels of methylation were observed in both CCGG and TCGA sites. Higher methylation levels were observed at the TCGA site when compared to the CCGG site (*p* < 0.0001, Mann-Whitney U-test), except in the adults I stage (*p* = 0.48). Statistically significant variations in methylation levels at the TCGA site between different ontogenetic stages were found (*p* < 0.01, Kruskal-Wallis test). We also detected 5-hydroxymethylation of CCGG sites, the highest levels being in the adults I stage. Variation in 5-hmC levels between different ontogenetic stages was significant as well (*p* < 0.01).

In selected regions of *Ephx1* and *Cand2* genes, methylation levels were much lower than in the *Cox4* gene, especially at CCGG sites, and levels of 5-hydroxymethylation were much higher. However, other tendencies were exactly the same: higher methylation was observed in TCGA sites when compared to CCGG sites (*p* < 0.01 for both genes and all developmental stages, Mann-Whitney U-test) and significant variation between developmental stages was found for methylation at the TCGA site (*p* < 0.01 for both genes, Kruskal-Wallis test). Mehylation at CCGG sites was low (actually at the lower limit of detection) and did not differ between different developmental stages. Again, variation in 5-hmC levels between different ontogenetic stages was significant for both *Ephx1* and *Cand2* genes (*p* < 0.01).

Thus, our analysis showed that both 5-mC and 5-hmC are present in gene bodies of three analyzed genes, and higher methylation levels are consistently found at TCGA sites when compared to CCGG sites. Since levels of both 5-mC and 5-hmC are changing during ontogeny of *D. pulex*, it may indicate that these epigenetic modifications are actively involved in gene expression of this crustacean.

The currently published *D. pulex* genome was sequenced using the Sanger sequencing method [11]. Here we describe the first attempt to assemble the genome using new generation sequencing data. For this we sequenced the genome using the Illumina MiSeq platform. The sequencing resulted in 1.4 M pair-ended reads and 1.7 G sequenced bases. This was assembled to 17,338 individual contigs with N50 of 10,601. All these contigs presented ~54% of the currently published *D. pulex* reference genome. The data on the quality of the assembly is given in Table 3. Detailed analysis showed that the *D. pulex* used in this study exhibited a lot of difference at the nucleotide level when compared with a published reference genome. The total identity for the sequenced part was only 93.6% between the two clones suggesting large differences in *D. pulex* of different populations.

It is well known that, morphologically, *D. pulex* is almost indistinguishable from the relative species *D. pulicaria* when comparing females [40]. However, in our samples only 6% of the reads mapped to the *D. pulicaria* genome, and most of the remaining reads mapped to the genome of *D. pulex.* Thus, it is obvious that mismatches between the reference genome and our sequenced *Daphnia* genome could not be attributed to incorrect identification of the species.

**Table 3:** Comparison of basic quality measures of assembled and reference genomes of *Daphnia pulex*.

	Assembled Genome		Reference Genome [11]	
Assembly	Contigs	Contigs used for peak calling and annotation	Contigs	Scaffolds
# contigs	17338	5572	19008	5191
Total length	85410271	66080228	158604366	197261574
GC (%)	40.74	41.08	40.76	40.77
N50	10601	13511	49250	642089
# N's per 100 kbp	0	0	1.33	19582.65

One explanation could be that different populations of *D. pulex* exhibit different genomes. The reference genome is sequenced using *D. pulex* clone “The chosen one” which is established in the USA. It is known that different clones of *D. pulex* from different continents have quite significant differences in their DNA sequences and that mutation rates of the mitochondria genome are one of the highest among different species [41].

**Figure 4:**
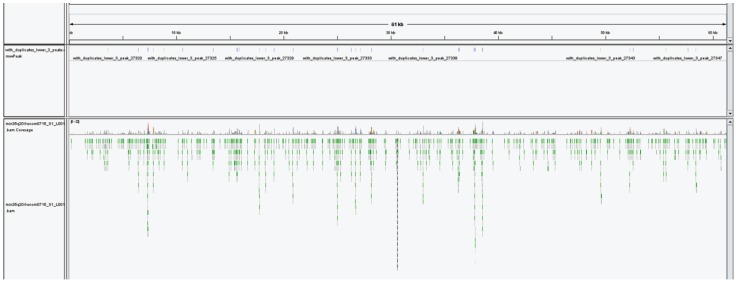
A representative region of the of assembled *D. pulex* genome contigs with mapped reads from one 5-hmC-enriched library (snapshot from IGV [42]). The upper lane shows detected peaks. The lower lanes shows individual reads along with coverage map.

To further investigate 5-hmC distribution over the *D. pulex* genome, we performed 5-hmC enrichment and sequenced the enriched fraction using next generation sequencing and non-enriched library (input) as a control. Two technical repeats were analyzed for enriched libraries. The sequencing resulted in ~17 M pair-end 75 bp reads for input and 40 M reads for enriched DNA (two runs). After alignment of the input library to the assembled genome, we did not manage to have even coverage; thus, for peak calling we used only the enriched library and detected more than 27,000 peaks (Figure 4). Although this is quite a risky approach, we managed to get valuable information as the detected peaks were not distributed randomly. Furthermore, we investigated the distribution of the peaks on the genomic elements to find if the 5-hmC were concentrated in biologically significant genomic regions. Firstly, we identified functional genomic elements on the newly assembled genome using annotation data from the reference *D. pulex* genome. Secondly, the peaks were assigned to different elements and density per kilobase of the sequence was calculated (Table 4).

**Table 4:** Analysis of 5-hmC enrichment over *D. pulex* functional elements. Tags denote number of locations where 5-hmC was found to be enriched. Tags/Kb—density of peaks per kilobase of analyzed sequence in a given genomic element. CDS: coding sequence site; TES: transcription end site; TSS: transcription start site.

Group	Total_Bases	Tag_Count	Tags/Kb
CDS_Exons	17597397	10651	0.61
Introns	17376020	4652	0.27
TSS_up_1 kb	12615965	1012	0.08
TSS_up_5 kb	35083848	2304	0.07
TSS_up_10 kb	57615402	2585	0.04
TES_down_1 kb	11356574	1191	0.10
TES_down_5 kb	31789833	2351	0.07
TES_down_10 kb	53166077	2506	0.05

This analysis clearly showed that 5-hmC was located not randomly over the *D. pulex* genome but had a strong preference to gene body sequences with exons having highest level of enrichment (Table 4). Moreover, such 5-hmC distribution is already described in other eukaryotic organisms such as mammals where 5-hmC is preferentially enriched over exonic sequences [43]. This finding suggests the idea that 5-hmC acts as a conservative regulatory element on gene bodies over the different organisms.

This result was the first demonstration of 5-hmC presence in *D. pulex* and it also confirms the dot-blot and qPCR data where we found that *D. pulex* DNA have 5-hmC in their genome.

**Figure 5:**
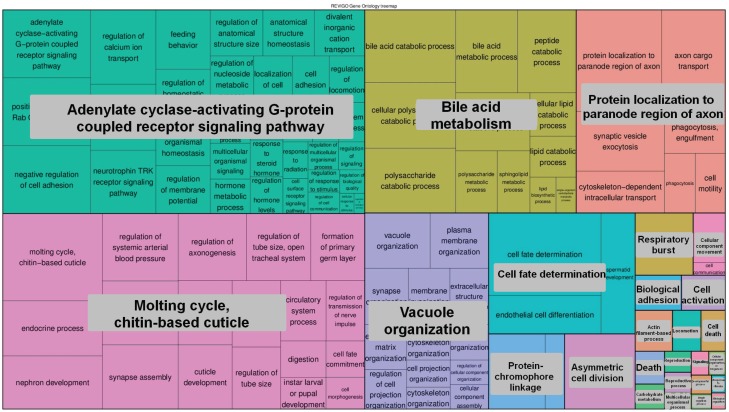
The GO terms (biological process) that were enriched for genes prone to 5-hmC modification. The areas of rectangles are proportional to the relative increase in frequency of GO terms comparing genes that had 5-hmC related peaks to genes that completely lacked them. The corresponding quantitative data and a full list of GO terms can be found in Supplementary File S1.

**Figure 6:**
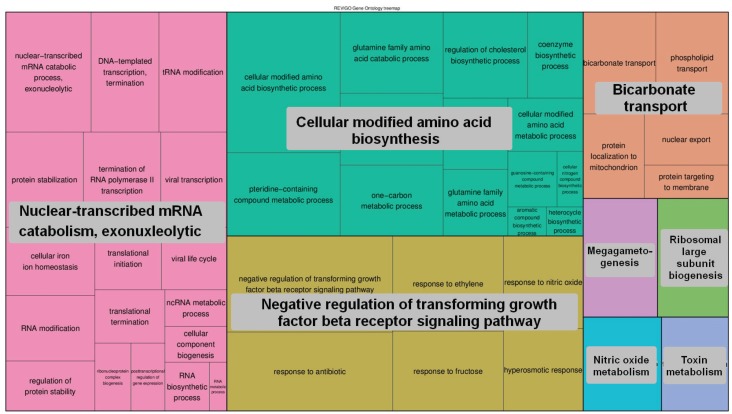
The GO terms (biological process) that were enriched for genes void of 5-hmC. The areas of rectangles are proportional to the relative increase in frequency of GO terms comparing genes that completely lacked 5-hmC-related peaks to genes that had them. The corresponding quantitative data and a full list of GO terms can be found in Supplementary File S2.

**Figure 7:**
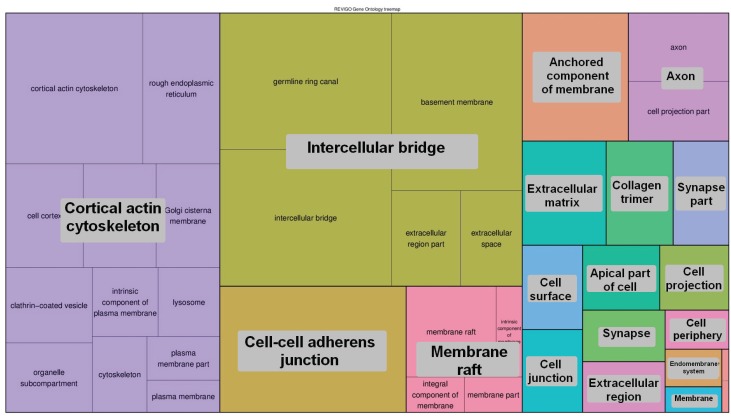
The GO terms (cellular components) that were enriched for genes prone to 5-hmC modification. The areas of rectangles are proportional to the relative increase in frequency of GO terms comparing genes that had 5-hmC-related peaks to genes that completely lacked them. The corresponding quantitative data and a full list of GO terms can be found in Supplementary File S3.

**Figure 8:**
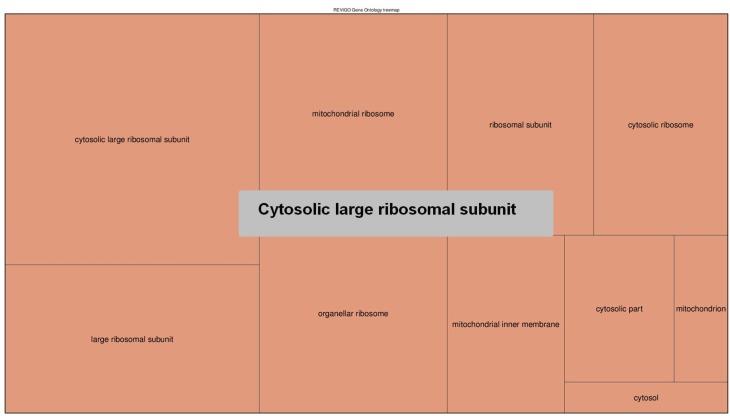
The GO terms (cellular components) that were enriched for genes void of 5-hmC. The areas of rectangles are proportional to the relative increase in frequency of GO terms comparing genes that completely lacked 5-hmC-related peaks to genes that had them. The corresponding quantitative data and a full list of GO terms can be found in Supplementary File S4.

For functional analysis we selected two groups of genes: (a) 1663 genes having exons completely void of peaks; (b) 2121 genes having a density of peaks across the gene body equal or higher than 10 PPKM. The detected enrichment of GO terms was clusterized and presented in Figure 5, Figure 6, Figure 7 and Figure 8. Quantitative data and a full list of GO terms can be found in Supplementary Files S1–S4. Our analysis suggests that 5-hmC abundance is associated with genes that are involved in many biological processes and notably in the adenylate cyclase-activating G-protein-coupled receptor signaling pathway and molting cycles. In addition, many genes with 5-hmC are involved in morphogenesis and, interestingly, in cell fate determination. On the other hand, the genes that lack 5-hmC tend to be involved in the regulation of the transforming growth factor beta (TGF-β) receptor signaling pathway and in many mRNA-related processes.

Quite an interesting discovery is the association between the low 5-hmC level and the TGF-β receptor signaling pathway, since it has been previously shown that TGF-β regulates DNA methyltransferase expression [44] in prostate cancer and TGF-β induces global changes in DNA methylation in ovarian cancer cells [45]. Therefore, it is very likely that TGF-β plays an important role in DNA epigenetic modifications in invertebrates as well. Additionally, it is evident from Figure 8 that genes lacking 5-hmC modifications mainly encode ribosomal proteins. It is quite interesting to note that other studies on mouse brain [46] indicated that neuron-specific 5-hmC-marked genes were enriched among genes coding ribosomal proteins. Thus, again, a lack of 5-hmC in genes of ribosomal proteins may be a general feature of eukaryotic organisms.

## 4. Conclusions

For the first time, we were able to demonstrate the presence of both 5-mC and 5-hmC in the genome of *Daphnia pulex*. Our analysis showed that both 5-mC and 5-hmC are present in gene bodies of three analyzed genes. It was also discovered that 5-hmC distribution across the genome is not random: in exons it is twice as frequent as in introns. Together with the results of the functional analysis of genes with no 5-hmC and a high number of peaks with 5-hmC, it may indicate that this epigenetic modification could act as a conservative regulatory element on gene bodies over the different eukaryotic organisms.

## Supplementary Files

Supplementary File 1
